# 0918. Grey matter perfusion is preserved during normotensive hypovolaemia, despite reductions in total cerebral blood flow: evidence of local, pressure independent, intra-cerebral vascular autoregulatory response

**DOI:** 10.1186/2197-425X-2-S1-O26

**Published:** 2014-09-26

**Authors:** SC Beards, JR Cain, LM Parkes, A Jackson

**Affiliations:** University Hospital of South Manchester, Acute Intensive Care Unit, Manchester, UK; University of Manchester, Wolfson Molecular Imaging Centre, Manchester, UK

## Introduction

We have recently demonstrated, using MRI measurements, that normotensive hypovolaemia (NTH) is associated with correlated decreases in cerebral blood volume flow (CBVF) and cardiac output (CO). This is in disagreement with previous studies using trans-cranial Doppler measurements.

## Methods

Eleven healthy volunteers (20-31 years; 8-male 3-female) underwent MRI studies with a lower body negative pressure (LBNP) chamber. Imaging included quantitative phase contrast angiographic (PCA) measurements of blood volume flow in the carotid and vertebral arteries and ASL of the whole brain. An ASL sequence followed by PCA acquisition was performed at rest (control) and under -20mmHg LBNP. ASL imaging used STAR labeling collected at 4 inversion times: 800ms, 1200ms, 1600ms and 2000ms. PCA acquisition was collected using 2D cine phase-contrast images. Throughout scanning the subjects pulse and blood pressure were monitored. ASL images were analysed using in-house code assuming a single blood compartment model^1^. Control and labelled images were subtracted and a two-parameter fit for bolus arrival time (BAT) and perfusion was performed on a voxel by voxel basis, producing perfusion and BAT maps. Perfusion was calculated with units ml/100ml/min. Automated tissue segmentation masks were created from aligned T1 images applied to co-registered perfusion and BAT maps for both cortical and sub-cortical grey matter structures.

## Results

**Figure 1:**
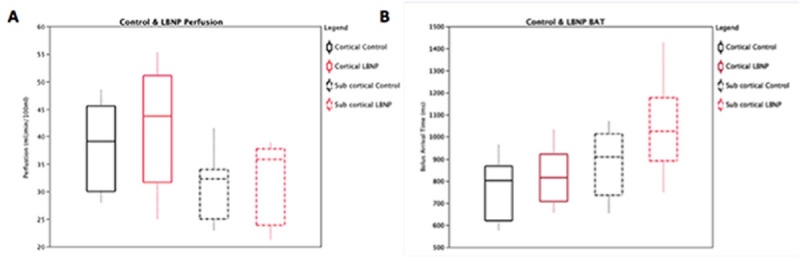
Figure 1

During -20mmHg LBNP, CBVF was reduced by 0.5 l/min, blood pressure remained constant and pulse was raised by 7 bpm. There was no difference between ASL grey matter perfusion values (mean 39.2ml/100ml/min and 42.3ml/100ml/min respectively) or sub-cortical grey matter perfusion values (31.3ml/100ml/min and 33.1ml/100ml/min) between control and -20mmHg (Fig 1A). BAT was significantly delayed (p< 0.05) during -20mmHg LBNP compared to control in both the cortical (782ms and 831ms) and sub-cortical grey matter (896ms and 1033ms); Fig 1B).

In the presence of constant perfusion this indicates regional vasodilatation. Comparing the sub-cortical structures, the hippocampus (19.5%) caudate (21%) and putamen (28%) showed the greatest % increase in BAT during -20mmHg LBNP.

## Conclusions

In young healthy individuals NTH induces reductions in CBVF which are compensated by vasodilatation of small vessels in normal grey matter, preserving grey matter perfusion despite reductions in total cerebral blood flow. This presumably occurs at the expense of white matter blood flow although that could not be measured with the current methodology. These results suggest a local, intra-cerebral autoregulation mechanism, independent of perfusion pressure, capable of preserving grey matter blood flow in the face of decreasing minute volume cerebral blood flow.
